# Enhanced anticancer activity of DM1-loaded star-shaped folate-core PLA-TPGS nanoparticles

**DOI:** 10.1186/1556-276X-9-563

**Published:** 2014-10-09

**Authors:** Xiaolong Tang, Yong Liang, Yongqiang Zhu, Shiyu Cai, Leilei Sun, Tianyi Chen

**Affiliations:** 1 Stem cell Engineering Research Center, School of Medical, Anhui University of Science & Technology, Huainan 232001, China; 2 The State Key Laboratory of Virology, Life Sciences College, Wuhan University, Wuhan, Hubei 430072, China; 3 Clinical Laboratory, The Affiliated Huai’an Hospital of Xuzhou Medical College, Huai’an 223002, China; 4 Department of Medical Genetics, Tongji Medical College, Huazhong University of Science and Technology, Wuhan, Hubei 430074, China; 5 Northeastern University, Boston, MA 02115, USA

**Keywords:** Emtansine (DM1), Targeting nanoparticles, Drug delivery

## Abstract

The efficient delivery of therapeutic drugs into interested cells is a critical challenge to broad application of nonviral vector systems. In this research, emtansine (DM1)-loaded star-shaped folate-core polylactide-d-α-tocopheryl polyethylene glycol 1000 succinate (FA-PLA-TPGS-DM1) copolymer which demonstrated superior anticancer activity *in vitro*/*vivo* in comparison with linear FA-PLA-TPGS nanoparticles was applied to be a vector of DM1 for FR^+^ breast cancer therapy. The DM1- or coumarin 6-loaded nanoparticles were fabricated, and then characterized in terms of size, morphology, drug encapsulation efficiency, and *in vitro* drug release. And the viability of MCF-7/HER2 cells treated with FA-DM1-nanoparticles (NPs) was assessed. Severe combined immunodeficient mice carrying MCF-7/HER2 tumor xenografts were treated in several groups including phosphate-buffered saline control, DM1, DM1-NPs, and FA-DM1-NPs. The antitumor activity was then assessed by survival time and solid tumor volume. All the specimens were prepared for formalin-fixed and paraffin-embedded tissue sections for hematoxylin-eosin staining. The data showed that the FA-DM1-NPs could efficiently deliver DM1 into MCF-7/HER2 cells. The cytotoxicity of DM1 to MCF-7/HER2 cells was significantly increased by FA-DM1-NPs when compared with the control groups. In conclusion, the FA-DM1-NPs offered a considerable potential formulation for FR^+^ tumor-targeting biotherapy.

## Background

Biodegradable nanoparticles served as drug carriers that could provide an ideal solution to most of the problems presented by anticancer drugs [1,2]. When compared with standard administration, drug incorporation into nanoparticles shows a variety of advantages such as high stability, protection of incorporated labile drugs from degradation, controlled and sustained drug release, increased drug therapeutic efficacy, and reduced side effects [3,4]. Polymers, including star-shaped cholic acid-core polylactide-d-α-tocopheryl polyethylene glycol 1000 succinate (PLA-TPGS) copolymer, PLA-TPGS-b-poly(ϵ-caprolactone-ran-lactide) (TPGS-b-(PCL-ran-PLA)) copolymer, and other structure copolymers, have been used to develop drug-loaded nanoparticles [5,6].

Similar to vinca alkaloids, maytansine and its analogs emtansine (DM1) which are microtubule-targeted drugs binding to tubulin at the vinca binding site can depolymerize microtubules and arrest cells in mitosis, resulting in a powerful anti-mitotic activity [7-10]. Maytansine displays almost 100 times higher cytotoxicity in cells than the vinca alkaloids [11,12]. Maytansine is effective against breast cancer, lung carcinoma, and murine melanocarcinoma solid tumors and has an antileukemic activity against lymphocytic leukemia *in vivo*[13]. The microtubule-targeted antiproliferative activity of maytansine/maytansinoids was substantiated in a screening of 60 human cancer cell types by the U.S. National Cancer Institute [13]. Although maytansine and DM1 efficiently inhibits microtubule assembly and then kills cancer cells, they demonstrated a small therapeutic window in human clinical trials [13,14]. The limitation of maytansine and DM1 is their systemic toxicity such as neurotoxicity and gastrointestinal tract adverse reaction [14,15]. Nevertheless, the powerful cytotoxicity of maytansinoids would make them valuable for anticancer therapies if tissue-specific drug delivery approaches are employed.

To achieve more active targeting, maytansinoids-loaded nanoparticle surface could be immobilized by functional molecules which can recognize and adhere to biomarkers on the surface of target cancer cells. Among targeting agents directed to the membrane-bound and tumor-associated receptors, folate (FA) has been widely utilized for targeted delivery of drug-loaded nanoparticles into FR^+^ tumor cells as a ligand for a folate receptor (FR) overexpressed in a wide range of tumors including those of the ovary, brain, kidney, breast, and myeloid [16-18]. Recently, it has been reported that FA-mediated delivery of drug-loaded nanoparticles can enable binding, promote uptake, and increase cytotoxicity to cancer cells *in vitro*/*vivo*[19-22].

In the present study, DM1-loaded-core PLA-TPGS copolymer nanoparticles (DM1-NPs) immobilized with FA which can enhance tumor cell-targeting ability were constructed. Characterization of the FA-modified DM1-loaded-core PLA-TPGS copolymer nanoparticles (FA-DM1-NPs), including size and zeta potential, was carried out. And drug entrapment efficiency, drug loading efficiency, and release properties *in vitro* were also tested. The targeting effect of FA-DM1-NPs was investigated *in vitro* through the uptake of fluorescent nanoparticles by FR^+^ MCF-7/HER2 cells. The results demonstrated that the FA-DM1-NPs and the stable DM1 methyl thioether derivative (S-DM1) could inhibit cell proliferation, arrest the mitotic process, and induce apoptosis in association with suppression of microtubule dynamic stability.

## Methods

### Material

Human breast adenocarcinoma cell line MCF-7/HER2(ER^+^, HER2-HIGH) was obtained from the American Type Culture Collection (ATCC; Rockville, MD, USA). TPGS, 4′-6′-diamino-2-phenylindole (DAPI), and PLA (*M*_w_ approximately 25,000) were purchased from Sigma-Aldrich (St. Louis, MO, USA). DM1 and S-DM1 was commercialized by Genentech. FA-PLA-TPGS copolymers (*M*_w_ approximately 23,000) were obtained from the Graduate School at Shenzhen, Tsinghua University. Antibodies against caspase-3/-8/-9 were obtained from Cell Signaling Technology (Beverly, MA, USA). Antibodies against Bcl-2 were obtained from Santa Cruz Biotechnology (Santa Cruz, CA, USA). All chromatographic solvents were of high-performance liquid chromatography (HPLC)-grade quality. And all other chemicals used were of the highest grade commercially available.

### Fabrication of DM1-loaded nanoparticles

A modified nanoprecipitation method was used to entrap DM1 into the FA-PLA-TPGS NPs [23,24]. Briefly, a pre-weighed amount of DM1 drug powder and 100 mg of FA-PLA-TPGS copolymer were dissolved in 8 mL of acetone by vortexing and sonication. The resulting nanoparticle suspension was stirred at room temperature overnight to remove acetone completely. The nanoparticle suspension was centrifuged at 25,000 rpm for 15 min, and then washed 2 to 3 times to remove the emulsifier and unloaded drug. This mixture was dropwise added into 100 mL 0.03% TPGS aqueous solution under stirring. In the end, the dispersion was lyophilized 48 h for further use. DM1-loaded PLA-TPGS NPs (DM1-PLA-TPGS-NPs) coumarin 6-loaded star FA-PLA-TPGS-NPs (coumarin 6-NPs), and linear FA-PLA-TPGS nanoparticles used as control were fabricated in a similar manner.

### Characterization of DM1-loaded nanoparticles

#### *Size, surface charge, and morphology of the NPs*

The nanoparticle size and zeta potential were determined by Malvern Mastersizer 2000 (Zetasizer Nano ZS90, Malvern Instruments Ltd., Malvern, Worcestershire, UK). All measurements were measured at room temperature after equilibration for 10 min. Average size and zeta potential of different NPs were analyzed using a dynamic light-scattering detector (Zetasizer Nano ZS90, Malvern Instruments Ltd., Malvern, Worcestershire, UK). The data were obtained with the average of three measurements.

The surface morphology of nanoparticles was examined by a field emission scanning electron microscopy (FESEM, JEOL JSM-6301 F, JEOL, Tokyo, Japan). To prepare samples for FESEM, the nanoparticles were fixed on the stub by a double-sided sticky tape and then coated with a platinum layer by JFC-1300 automatic fine platinum coater (JEOL, Tokyo, Japan). Morphological examination of FA-PLA-TPGS-NPs was performed using transmission electron microscopy (H600, Hitachi, Tokyo, Japan).

#### *Drug content and entrapment efficiency*

To determine the contents of drug loading (LC) and entrapment efficiency (EE) of the FA-DM1-NPs, a predetermined amount of nanoparticles were dissolved in 1 mL methylene dichloride under vigorous vortexing. The solution was transferred to 5 mL of mobile phase consisting of acetonitrile and deionized water (50:50, *v*/*v*). A nitrogen stream was introduced to evaporate the methylene dichloride for approximately 20 min. And then a clear solution was obtained for high-performance liquid chromatography (HPLC) analysis (LC 1200, Agilent Technologies, Santa Clara, CA, USA). A reverse-phase C_18_ column (250 mm × 4.6 mm, 5 μm, C_18_, Agilent Technologies, Santa Clara, CA, USA) was used at 25°C. The flow rate of mobile phase was 1 mL/min. The column effluent was detected using a UV detector at *λ*_max_ of 227 nm. The measurement was performed in triplicate. Drug loading and encapsulation efficiency of the drug-loaded nanoparticles were calculated according to the following equations, respectively.

$$\begin{array}{c} LC\left(\%\right)=\;\left(\text{Weight}\;\text{of}\;DM1\;\text{in}\;\text{the}\;\text{nanoparticles}\right)/\\ \;\left(\text{Weight}\;\text{of}\;\text{the}\;\text{nanoparticles}\right)\times 100\% \end{array}$$

$$\begin{array}{c} EE\left(\%\right)=\;\left(\text{Weight}\;\text{of}\;DM1\;\text{in}\;\text{the}\;\text{nanoparticles}\right)/\\ \;\left(\text{Weight}\;\text{of}\;\text{the}\;\text{feeding}\;DM1\right)\times 100\% \end{array}$$

Briefly, 10 mg of FA-DM1-NPs were introduced into Eppendorf tubes and dissolved in 1 mL acetonitrile and diluted by 0.1 M citric acid. Meanwhile, the amount of DM1 in the solution was determined by HPLC.

#### *Drug-release assay study*

The *in vitro* release profile of DM1 from FA-DM1-NPs was determined by measuring the residual amount of DM1 presented in the nanoparticles [25]. In brief, 5 mg of accurately weighted lyophilized nanoparticles (FA-DM1-NPs) were put into a centrifuge tube and redispersed in 8 mL phosphate buffer solution (PBS, containing 0.1% *w*/*v* Tween 80, pH 7.4). The tube was put into an orbital shaker water bath and vibrated at 130 rpm at 37°C. At certain time intervals, the tube was taken out and centrifuged at 25,000 rpm for 15 min. The supernatant was then transferred into a glass test tube for HPLC analysis. The pellet was resuspended in 8 mL fresh PBS buffer and put back into the shaker bath for subsequent determination. The accumulative release of DM1 from nanoparticles was plotted against time.

### Evaluation of the biological function of FA-DM1-NPs

#### *Cellular uptake of FA-coumarin 6-NPs*

In this research, coumarin 6 served as a model control molecule, which can be entrapped in star-shaped FA-PLA-TPGS NPs for qualitative and quantitative studying on cellular uptake by MCF-7/HER2 cells. Cells incubated with 250 μg/mL coumarin 6-NPs at 37°C for determined time (12, 24, and 36 h) were rinsed with cold PBS solution three times, and then fixed by methanol for 25 min. Cells were stained with DAPI for 30 min to display the nuclei and rinsed twice with a PBS solution [26,27]. Tumor cells were observed by using confocal laser scanning microscopy (CLSM; LSM 410, Zeiss, Jena, Germany) with an imaging software.

#### *In vitro cytotoxicity of FA-DM1-NPs*

MCF-7/HER2 cells were seeded in 96-well plates at the density of 5 × 10^3^ viable cells per well in 100 μL of culture medium and incubated overnight. Cells were then treated with various suspension concentrations ranging from 0.001 to 10.0 μg/mL of DM1 and FA-DM1-NPs for 48 and 72 h at 37°C in a CO_2_ incubator. At certain time intervals, the nanoparticles were replaced with DMEM containing MTT (5 mg/mL) and cells were then incubated for an additional 4 h. MTT was aspirated off, and DMSO was added to each well to solubilize the formazan crystals formed in viable cells. The absorbance of each well was recorded at 570 nm using a 96-well microplate reader. Viability of untreated cells was set at 100%, and absorbance of wells with medium and without cells was set as zero. All of the results were from at least triplicate experiments. The inhibitory concentration IC_50_, the drug concentration at which cell growth was inhibited by 50% relative to untreated control cells, was calculated by curve fitting of the cell viability vs. drug concentration data [28,29].

#### *In vivo assay of drug sensitivity*

The Administrative Committee on Animal Research in the Anhui University of Science and Technology approved all the protocols for the proposed human breast cancer cell lines and animal experiments. Female BALB/c nude mice of 15 to 20 g and 4 to 5 weeks old were purchased from the Institute of Laboratory Animal Sciences, Chinese Academy of Medical Science. MCF-7/HER2 cells in the medium were inoculated subcutaneously to mice in the amount of 2 × 10^6^ cells per mouse at the right axilla, and the subcutaneous tumor growth in each mouse was monitored. The length and width of tumors were determined by a vernier caliper, and the tumor volume (*V*) was calculated as *V* = *d*^2^ × *D*/2, where d and D are the shortest and the longest diameter of the tumor in mm, respectively [30]. When the tumor volume reached approximately 50 mm^3^ (set as the 0 day), treatments were performed. The mice were randomly divided into three groups (each group has 10 mice, *n* =10). The two formulations of DM1, i.e., the drug FA-DM1-NPs and Kadcyla®, were injected via intra-tumoral at a single dose of 10 mg DM1/kg in PBS on days 10, 13, and 16. PBS served as the control. Mice were sacrificed by decapitation 30 days after treatment. The terminal tumor weight (mg) was determined and applied to evaluate the antitumor effects.

#### *Immunohistochemistry*

Xenograft tumor tissues were fixed in phosphate-buffered formalin, embed in paraffin, and cut in 4 μm thickness. Sections were deparaffinized and stained with hematoxylin-eosin (H&E). For immunohistochemistry (IHC), tissue sections were covered with 10 mM sodium citrate buffer, pH 6.0, and heated in a convection steamer for 1 h. After being blocked in 5% normal goat blocking serum for 30 min, it was incubated with primary antibody for 1 h, and next as IHC operation.

#### *Fluorescence in situ hybridization (FISH)*

*Her2* gene copy numbers were determined by using the dual-color fluorescence *in situ* hybridization (FISH). After trypsinization and washing with PBS, the cells were fixed with methanol-acetic acid (3:1) and air-dried on slides. A bacterial artificial chromosome (BAC) clone specific to *Her2* DNA was labeled with dUTP-FITC (Fermentas, Burlington, Canada) and the chromosome 17 centromere probe (p17H8) was labeled with chromatide Alexa Fluor 594-5-dUTP (Invitrogen, Carlsbad, CA, USA) using nick translation, and FISH was performed as described previously [31].

#### *Western blot*

The expression levels of procaspase-3/-8/-9 and Bcl-2 in the breast cancer cells treated with S-DM1 or FA-DM1-NPs were analyzed by Western blot. Protein was extracted and separated by SDS-PAGE and transferred to nitrocellulose membranes. After being blocked in 5% bovine serum albumin (*w*/*v*) at room temperature for 1 h, the membranes were rinsed and incubated at 4°C overnight with primary anti-total and procaspase-3/-8/-9, β-actin antibody (1:1,000). The membranes were then washed and incubated with secondary antibody (1:2,000 ~ 3,000) at room temperature for 1 h, developed with chemiluminescence ECL reagent (LumiGold, SignaGen, Gaithersburg, MD, USA), and exposed to Hyperfilm MP (GE Healthcare, Piscataway, NJ, USA). Tumor samples were lysed in RIPA buffer (50 mM Tris-HCl, pH 7.5, 150 mM NaCl, 5 mM EDTA, 0.1% SDS, 0.5% sodium deoxycholate, 1% NP-40, supplemented with complete Mini protease inhibitors) and equal amounts of protein were subjected to Western blotting analysis. The protein levels were normalized to β-actin.

### Statistical methods

All experiments were performed at least three times unless otherwise mentioned. Student’s *t* test statistical analysis was carried out with SPSS 13.0 software, with *p* <0.05 considered to indicate a significant difference.

## Results and discussion

### Size, surface morphology, zeta potential, and entrapment efficiency

**Figure 1:**
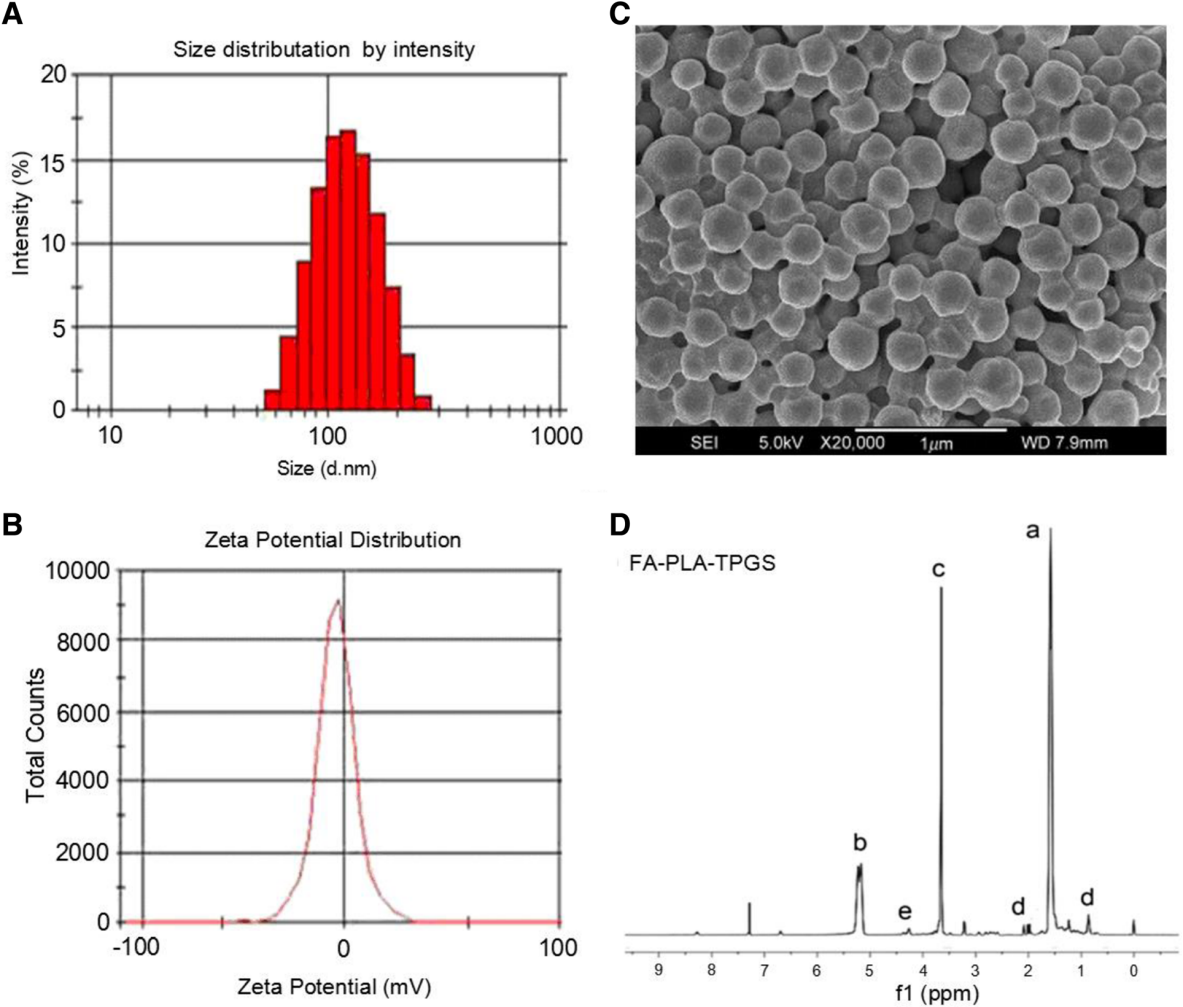
**The physical properties of the star-shaped FA-PLA-TPGS nanoparticles. (A)** Size distribution of the star-shaped FA-PLA-TPGS nanoparticles detected by DLS. **(B)** FESEM image of the star-shaped FA-PLA-TPGS nanoparticles. **(C)** Zeta potential distribution of the star-shaped FA-PLA-TPGS NPs. **(D)** Typical ^1^H NMR spectra of FA-PLA-TPGS copolymers.

Particle size and surface properties of the nanoparticles play a crucial role in drug-release kinetics, cellular uptake behavior, as well as *in vivo* pharmacokinetics and tissue distribution [24]. The particle size and size distribution of the FA-DM1-NPs were displayed in Table 1. According to PDI, ZP (mV), particle size (nm), LC (%) and EE (%) parameters, star-shaped folate-core PLA-TPGS copolymer nanoparticles displayed perfect advantages as an efficient drug-delivery vehicle. The physical properties of the FA-DM1-NPs were displayed in Figure 1. ^1^H NMR (CDCl 3): a (*δ* =1.61 ppm, LA repeating unit: -CHCH 3), b (*δ* =5.19 ppm, LA repeating unit: -CHCH 3), c (*δ* =3.65 ppm, TPGS repeating unit: -CH 2 CH 2 O-), d (*δ* =0.52 to 2.30 ppm, FA moiety: -CH- and -CH2-), and e (*δ* =4.41 ppm, terminal hydroxyl group of FA-PLA: -CHOH). The average hydrodynamic size of the FA-DM1-NPs is approximately 126.6 ± 3.5 nm in diameter, which is in the excellent size range for readily accumulating in tumor vasculature due to the enhanced permeation and retention effects [30,32].

**Table 1:** Characterization of DM1-loaded nanoparticles

**Polymer**	**Particle size (nm)**	**PDI**	**ZP (mV)**	**LC (%)**	**EE (%)**
PLA-TPGS-DM1	123.7 ± 3.9	0.199	-19.5 ± 2.6	8.55	76.01
FA-PLA-TPGS-DM1	126.6 ± 3.5	0.154	-12.0 ± 2.1	10.35	84.03

PDI, polydispersity index; ZP, zeta potential; LC, loading content; EE, entrapment efficiency. *n* =3.

### *In vitro* release profiles

**Figure 2:**
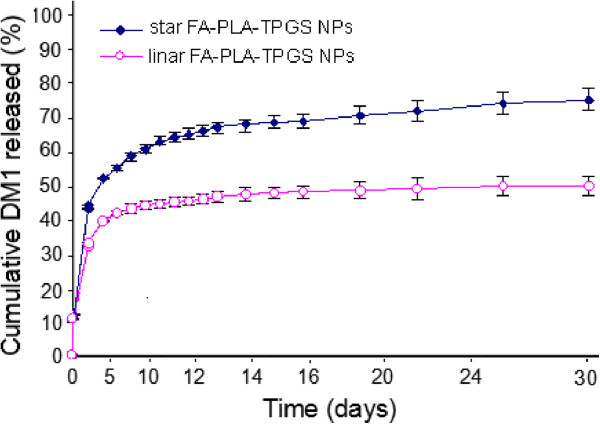
***In vitro *drug release of FA-DM1-NPs.** A rapid release was observed from 0 to 5 days, with a cumulative release percentage of 39.2% (linear FA-PLA-TPGS nanoparticles) and 53.4% (star FA-PLA-TPGS nanoparticles) in the first 5 days. Smooth slow release occurred between days 5 and 12, with a cumulative release percentage of 65.5% (star FA-PLA-TPGS nanoparticles) and 44.3% (linear FA-PLA-TPGS nanoparticles). During days 12 to 16, the release reached a plateau, with a cumulative release percentage of 70.7% at day 14.

The *in vitro* drug release profiles of the FA-DM1-NPs in PBS (pH 7.4) in the first 30 days were shown in Figure 2. The continuous release of drugs from the polymeric nanoparticles could occur either by diffusion of the drug from the polymer matrix or by the erosion of the polymer [33,34]. The initial burst release in the first 5 days was due to the drug encapsulated in the periphery of the nanoparticles, while the subsequent sustained release was predominantly attributed to the diffusion of the drug which was well entrapped in the core of nanoparticles. The DM1 release from the linear FA-PLA-TPGS nanoparticles and star FA-PLA-TPGS nanoparticles displayed an initial burst of 39.2 and 53.4% in the first 5 days, respectively. The accumulative DM1 release in the first 30 days was found in the following order: star FA-PLA-TPGS nanoparticles (65.5%) > linear FA-PLA-TPGS nanoparticles (44.3%). The star FA-PLA-TPGS nanoparticles displayed the fastest drug release, indicating that the star-shaped FA-PLA-TPGS copolymer was capable of displaying faster drug release than that of the linear FA-PLA-TPGS nanoparticles when the copolymers had the same molecular weight. Similar results can be found in the literature [24]. The generally sustained and controlled release profile of DM1 facilitates the application of nanoparticles for the delivery of anticancer drugs.

### Cellular uptake of fluorescent FA-PLA-TPGS nanoparticles

**Figure 3:**
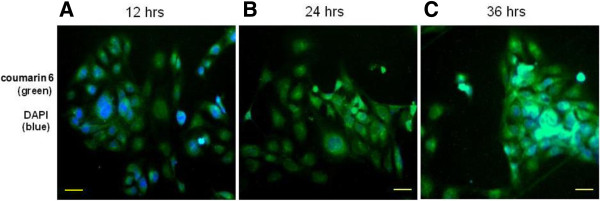
**CLSM images of MCF-7/HER2 cells after determined time of incubation with coumarin 6-NPs.** The coumarin 6-loaded nanoparticles were green, and the cell nuclei were stained by DAPI (blue). The cellular uptake was visualized by overlaying images obtained respectively from the EGFP filter and DAPI filter: **(A)** After 12 h incubation with coumarin 6-NPs, **(B)** after 24 h incubation with coumarin 6-NPs, and **(C)** after 36 h incubation with coumarin 6-NPs. *Scale bar* 10 μm.

It has been demonstrated that the therapeutic effects of drug-loaded nanoparticles depend on internalization and sustained retention of the nanoparticles by diseased cells [35,36]. The studies *in vitro* were capable of providing some circumstantial evidence to show the advantages of nanoparticle formulation over free drug. Coumarin 6 served as a fluorescent probe in an attempt to represent the drug in the nanoparticles for visualization and quantitative analysis of cellular uptake of the nanoparticles [37,38]. Figure 3 show the images of tumor cells after determined time (12, 24, and 36 h) of incubation with coumarin 6-loaded FA-PLA-TPGS nanoparticle dispersion in DMEM at the concentration of 250 μg/mL. It can be seen from this figure that, with the extension of the schedule, the green fluorescent in cells continuously enhanced, indicating that the fluorescent nanoparticles had been internalized into these MCF-7/HER2 cells.

### *In vitro* MCF-7/HER2 cells viability and sensitivity

**Figure 4:**
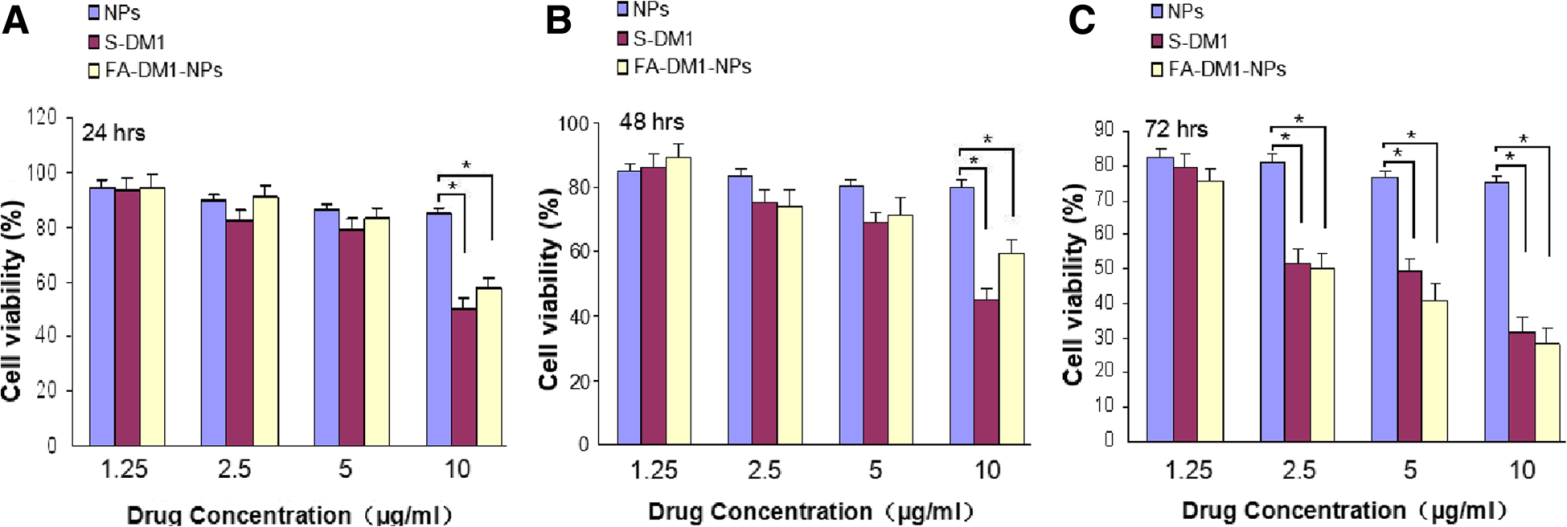
**MCF-7/HER2 cell viability of FA-DM1-NPs compared with that of S-DM1.** At equivalent S-DM1 dose concentration. **(A)** After 24 h incubation. **(B)** After 48 h incubation. **(C)** After 72 h incubation (*p <0.05, *n* =3).

**Figure 5:**
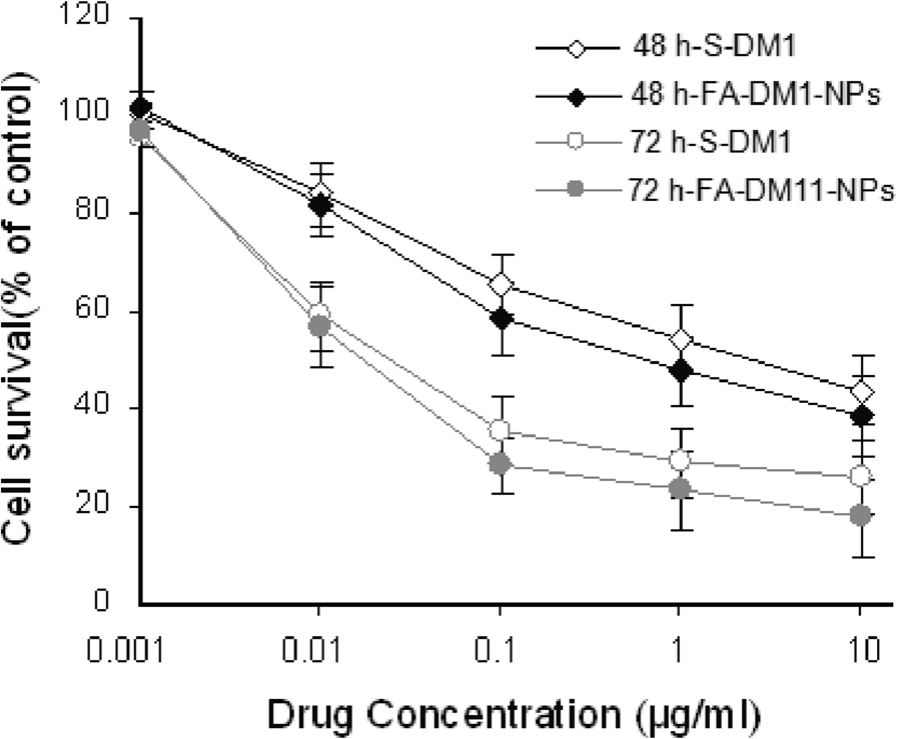
***In vitro *growth inhibitory effects of S-DM1 and FA-DM1-NPs on MCF-7/HER2 cells.** The anticancer effects of FA-DM1-NPs and S-DM1 were tested at concentrations of 0.001, 0.01, 0.1, and 1.0 and 10.0 μg/mL after treatment for 48 and 72 h, respectively. Both drug inhibited the growth of MCF-7/HER2 cells in a dose-dependent manner, but the effect on FA-DM1-NPs had no difference with that of S-DM1 in the concentration range of 0.01 to 10.0 μg/mL (*p* >0.05).

Human MCF-7/HER2 cells were applied to investigate the cytotoxicity of DM1-loaded nanoparticles. The S-DM1 was designed as the positive control. The different groups of nanoparticles were sterilized by gamma radiation. Figure 4 displayed the *in vitro* cell viability of FA-DM1-NPs at equivalent S-DM1 concentrations of 1.25, 2.5, 5.0, and 10.0 μg/mL. A quantitative colorimetric assay of MTT was used to determine the percentage of viable cells [30,39]. From Figure 4, it can be concluded that the cell suppression of S-DM1 and FA-DM1-NPs showed both dose- and time-dependent responses. The cell viability decreased steadily with increasing drug dose and incubation time, especially for FA-DM1-NPs. Although the growth inhibition efficacy of FA-DM1-NPs was stronger than that of S-DM1 in the concentration range of 0.1 to 10 μg/mL, there were no significant differences between FA-DM1-NPs and S-DM1 statistically (Figure 4). Next, we compared the effects of S-DM1 and FA-DM1-NPs on MCF-7/HER2 cell lines *in vitro*. After 48- and 72-h incubation, respectively, FA-DM1-NPs and S-DM1 inhibited the growth of MCF-7/HER2 cells in a dose-dependent manner. The growth inhibition of FA-DM1-NPs was stronger compared with that of S-DM1 in the concentration range of 0.1 to 10.0 μg/mL (*p* >0.05, *n* =3) (Figure 5). But statistically, there were no significant differences between FA-DM1-NPs and S-DM1.

### Effect of drug on the expression levels of apoptosis-related proteins

**Figure 6:**
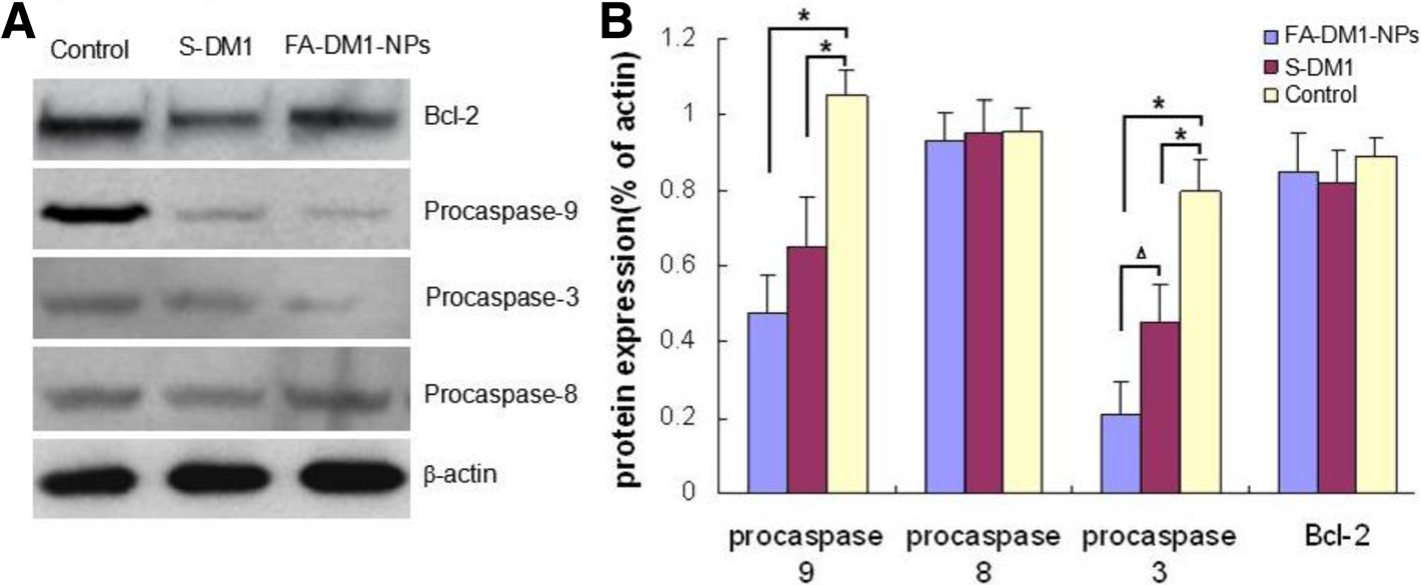
**Effect of FA-DM1-NPs and S-DM1 on expression levels of apoptosis-related proteins in MCF-7/HER2 cells.** Following the treatment of breast cancer cells with therapeutic concentrations of drugs (5.2 µg/mL S-DM1 and 10 µg/mL FA-DM1-NPs equivalent DM1 5.0 µg/mL, respectively), the expression levels of procaspase-3/-8/-9 and Bcl-2 were analyzed by Western blot **(A)** and Gel-Pro Analyzer 4.0 software **(B)**. **p <0.01, *p <0.05 vs. control. Results were representative of three independent experiments. ^Δ^*p* <0.05 vs. S-DM1 treatment group.

In order to study the effect of FA-DM1-NPs and DM1 on the expression levels of apoptosis-related proteins, treatment concentrations of drugs were administered to MCF-7 cells for 48 h. And Western blot analysis was conducted to investigate the expression levels of Bcl-2 and procaspase-3/-8/-9 proteins (Figure 6). It was found that following treatment with FA-DM1-NPs or DM1, the procaspase-3 and procaspase-9 protein expression levels significantly declined, while no significant differences were observed in the expression levels of procaspase-8 protein. Our data demonstrated that DM1 and FA-DM1-NPs-induced apoptosis may occur through the mitochondria-mediated caspase-3 and caspase-9 pathways.

### Effect of DM1 NPs on xenografts

**Figure 7:**
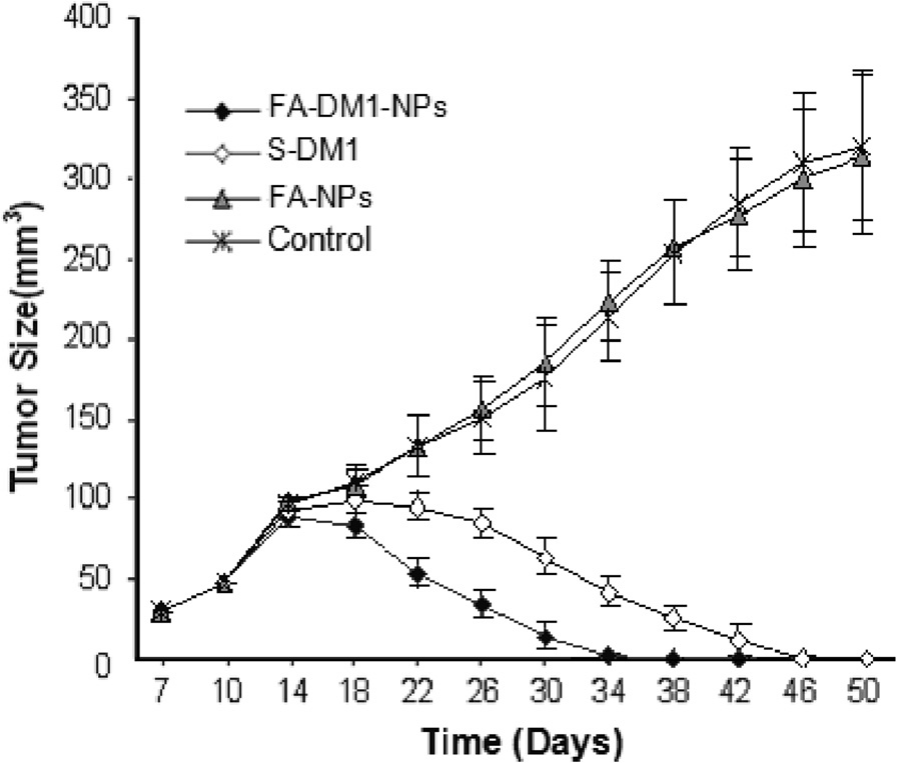
**Growth inhibitory effects of FA-DM1-NPs and S-DM1 on MCF-7/HER2 cells *in vivo*.** SCID mice with tumor cell xenografts were treated with PBS, FA-NPs (6 μg/g, i.v., *n* =10), FA-DM1-NPs (10 μg/g equivalent DM1 5 μg/g, i.v., *n* =10), or S-DM1 (5.2 μg/g equivalent DM1 5 μg/g, i.v., *n* =10). Drug administration was conducted on days 10, 13, and 16, respectively. FA-NPs had no effect on tumor growth. Tumor shrinkage was seen by DM1 from day 38. From day 34 onwards, all of the FA-DM1-NP-treated tumors were unmeasurable.

**Figure 8:**
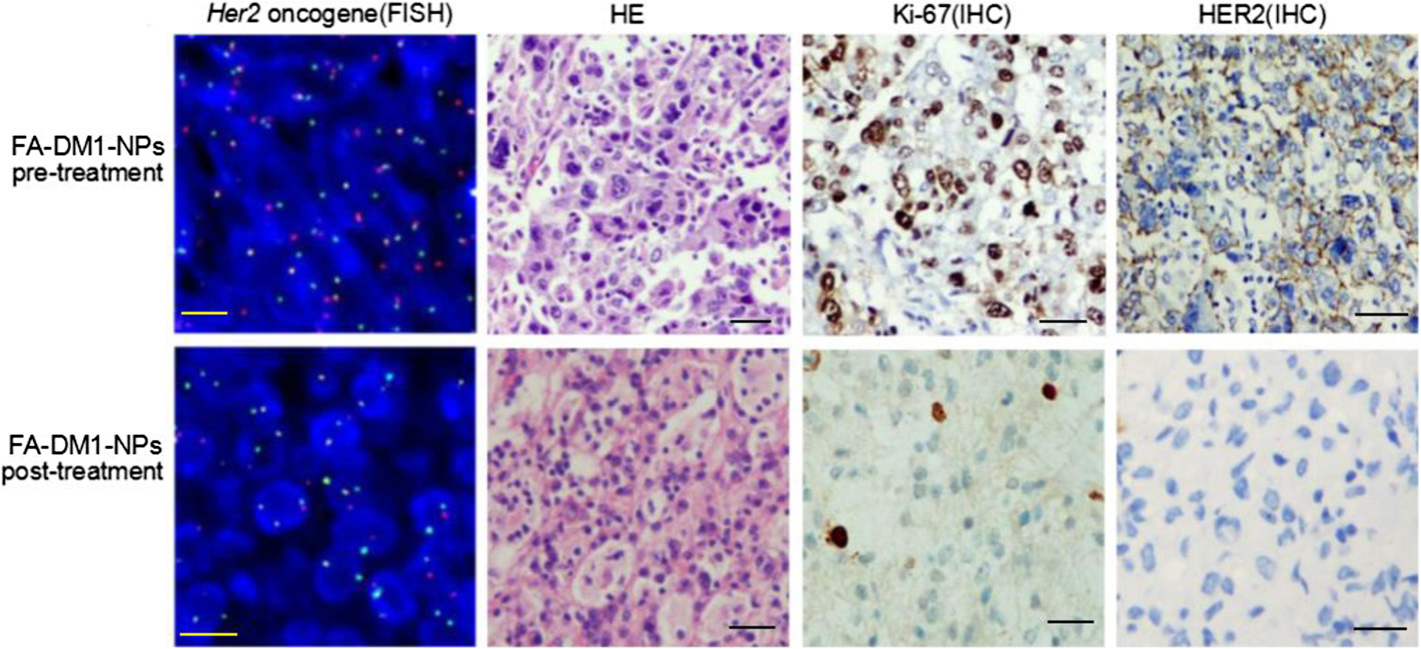
**Residual cancer cells in FA-DM1-NPs treated MCF-7/HER2 xenografts.** Residual cancer cell aggregates were found from the tumor samples in two of ten xenografted mice after FA-DM1-NP treatment. The level of oncogene *Her2* (FISH), tumor cell division phase index Ki-67 (IHC) positive ratio, and the expression level of HER2 (IHC) in the MCF-7/HER2 cells of the FA-DM1-NP post-treatment were lower compared with those of pre-treatment (×100). *Yellow scale bar* 10 μm and *black scale bar* 50 μm. The few surviving in the tumor tissue FA-DM1-NP treatment group after treatment, the level of oncogene *Her2*, tumor cell division phase index Ki-67, and the HER2 expression level of tumor cells were significantly reduced, even disappeared, which suggested that the existence of the typical MCF-7/HER2 tumor cells had significantly declined. This may be due to FA-DM1-NPs combining folate receptor (FR) of the tumor cells effectively to induce the FA-DM1-NPs internalize and release the entrapped drug DM1, and then play the biological effects of antitumor. These data suggested that FA-DM1-NPs can be more effectively enriched in FR^+^ MCF-7/HER2 tumor cells and have more effective antitumor as compare to S-DM1.

MCF-7/HER2 cells were capable of forming xenograft subcutaneous tumors in SCID mice in 7 days (*n* =40, mean tumor volume 30 ± 3 mm^3^). Thereafter, treatments with S-DM1 (*n* =10) or FA-DM1-NPs (*n* =10) were administered on days 10, 13, and 16. FA-NPs (*n* =10) was used as negative control agent, and PBS as control agent (*n* =10). A rapid tumor shrinkage was observed by S-DM1 from day 46 (Figure 7). Histologically, a pathological complete response was seen in five mice (5/10), and residual tumor cells in the other five (5/10) at the end of the experiment (day 50) (Figure 8). Overall, the effect of FA-DM1-NPs on MCF-7/HER2 tumor growth was much stronger than that of S-DM1. From day 34 onwards, almost all of the FA-DM1-NP-treated tumors were unmeasurable. Histologically, a pathological complete response was seen in eight mice (8/10) and residual tumor cells in the other two (2/10) at the end of the experiment (day 50) (*p* <0.05) (Figure 7). FA-NPs and PBS had no effect on tumor growth. It can be seen from Figure 7 that FA-DM1-NPs can effectively control MCF-7/HER2 xenograft tumor.

### Pathological analysis

Synergistic antitumor activities could be obtained by the use of combinations of S-DM1 and star FA-PLA-TPGS-NPs. As shown in Figure 8, residual tumor cells were found at the inoculation in two of ten samples (2/10) in the FA-DM1-NP treatment group. Tumor cell nuclear structure showed that cell division phase decreased significantly compared to pre- FA-DM1-NP treatment. The fraction of proliferating cells (confirmed by Ki-67 immunohistochemical staining) among tumor cells was small compared to pre-treatment (Figure 8). Confirmed by FISH, oncogene *Her2* was amplified in MCF-7/HER2 cells of the pre- and postdrug treatment. But the *Her2* level at pre-treatment was much higher than that at post-treatment (Figure 8). Tumor cell division phase index Ki-67 (IHC) positive ratio and the expression level of HER2 (IHC) in MCF-7/HER2 cells of the FA-DM1-NP post-treatment were lower compared with those of pre-treatment. HER2 is an excellent target for an antibody-drug conjugate as it is highly overexpressed on HER2-positive cancer cells. After binding to HER2, DM1 is internalized by endocytosis and degraded in lysosomes, causing the release of the active metabolite DM1 which inhibits *Her2*- and HER2-positive cancer cell division and induces cell death by blocking the spindle apparatus. So, after FA-DM1-NP pre-treatment, tumor cell division phase index-, *Her2* oncogene-, and HER2-positive cancer cells significantly reduced compared with FA-DM1-NP post-treatment. In conclusion, the FA-DM1-NPs offered considerable potential as an ideal candidate for *in vivo* drug delivery.

## Conclusions

There is no doubt that DM1 has provided significant clinical benefit in patients with breast cancer [36,37]. Nevertheless, primary (de novo) and secondary (acquired) resistance and side effects represent real clinical challenges [25,40]. FA-DM1-NPs showed high stability and desired surface properties in favor of cellular uptake. Meanwhile, FA-DM1-NPs can be targeted to FR^+^ cancer cells and improve DM1 concentration in the target tissues, reduce nonspecific distribution and side effects of DM1, inhibit tumor cell proliferation, and promote apoptosis of tumor cells in the effects of biological synergy, which has a potential to establish new treatment paradigms.

## Competing interests

The authors declare that they have no competing interests.

## Authors’ contributions

XLT and YL carried out the polymer synthesis, nanoparticle preparation, and cell studies. YQZ participated in the cell studies and the animal studies. SYC carried out the polymer characterization and nanoparticle characterization. LLS participated in the polymer synthesis and characterization. TYC conceived the study and participated in its design and coordination. All authors read and approved the final manuscript.
